# Clinical Cases

**Published:** 1890-04

**Authors:** F. L. Griffith

**Affiliations:** Edina, Missouri


### CLINICAL CASES.

F. L. Griffith, M. D., Edina, Missouri.

For over a year I have read that valuable journal The Homœopathic Physician; I have read it thoughtfully and with great interest. I cannot see how any pure homœopath can do without it, and the mongrel should take it in order to find out that he is not a homœopath, or anything else, though he poses serenely under the protecting wings of Homœopathy. He is proud of the name, it gives him prestige among intelligent people, but the poor fellow is too densely ignorant to know the first principles of a pure homœopathic prescription. "He uses Homœopathy till he finds it fails, then he resorts to *something else*," and he prescribes the *something else* about as intelligently as he did Homœopathy.

I will tell you, Dr. Mix-em-up, that you are a disgrace to our school, and I have heard intelligent, honorable allopaths say you also disgrace their school. Then where do you belong? Methinks I hear the poor honest old eclectic sigh heavily when he sees he has to receive you. I have been trying for the past few years to excuse the Mix-em-up doctors, but it is impossible, my charity is drawn out to the finest point, and contempt is now the most charitable feeling I have for them. They are sailing under false colors, and that which we try so hard to uphold is held responsible for their bungling work.

I am proud of the I. H. A., and hope some day to be a member of that grand organization. I would like to see them found a college in some convenient city (say Rochester, N. Y.) where nothing but the purest of Homœopathy shall be taught, and only members of the I. H. A. shall lecture in the institution; then would we have more pure homœopathic physicians.

Most of our colleges teach a very impure form of Homœopathy, consequently most of our doctors who become homœopaths must become such after leaving the colleges.

I believe any studious, intelligent homœopath will practice according to the law of Hahnemann.

All it requires is a thorough and impartial test. Oh! what beauty there is in it, and how grandly it fulfills our expectations!

Missouri has been a little backward in accepting Homœopathy, but we have now a few noble followers of the law, who are sowing the seed of truth, and ere long this will furnish abundant homes for true disciples of Hahnemann.

I would like to report a few clinical cases here, cases that were treated according to the *law:*

CASE I.—Mr. L—— æt. fifty-four, merchant and stock dealer; tall, slender, black hair and keen eye, very wealthy, but is a physical wreck from business anxiety; stomach revolts at the plainest kind of food. Distress in stomach after eating even a little; sour stomach. About a month ago was attacked violently with la grippe, and the wise old Quinine vendor was called in to assist in the killing. After six days of dosing, the family had about given up all hopes of his recovery, but as a last resort they called in the homœopath. I found him so weak he could scarcely speak. Stupefying headache, hot head, cold feet and hands, extremely nervous, entire inability to sleep night or day, except perhaps a moment at a time, then his eyes would be half open. Great burning at anus. Redness and burning at orifice of urethra. Ears very red. One dose Sulph.$^{200}$, dry, followed by S. L. in water every hour when awake. They did not have a chance to give a dose of the S. L. for nearly three hours, he having slept two and a half hours, and awoke feeling wonderfully better, and gradually improving; a few days later he was troubled with chills which were stopped with Nux-v.$^{30}$, three doses thirty minutes apart.

During all these days he had a violent cough, with profuse expectoration of thick, yellow, purulent material. One thing extremely peculiar about the cough was its regular periodicity, coming on about nine A. M., and stopping about one P. M. every day. He never coughed any except between those hours. I consulted Lippe's *Repertory* (as I do in all cases), and found on page 183, "Cough at same time every day, Lyc., Sabad." One day, about three hours after the worst paroxysm he had had, I gave one dose of Sabad$^{60}$, dry, followed by my friend S. L. Will you believe me, dear Hahnemannians, when I tell you he never coughed again? This was as great a surprise to me as to the patient and the other members of his household. The stopping of such a cough with so much expectoration between the paroxysms seems incredible.

I am indebted to Dr. A. A. Whipple, of Quincy, Ill., for valuable assistance in the treatment of this case.

CASE II.—Mr. N., æt. thirty-six years. Chill at seven A. M., every other day. Before chill, boneache and thirst; during chill thirst, headache, eyeache, aching all over; chill begins in back; nausea between chill and fever; fever lasts all day; extremely weak.

Patient had been suffering so much from chills for several days that some of my friends persuaded him to try Homœopathy. I was called and found him with the above symptoms, but with no faith whatever in me. He said, "Doctor, I don't believe your sugar will cure such chills as I have. I am going to give you one trial to please my friends, and if you fail, I shall use Quinine, although that drug nearly kills me." As I was to have but one trial with this gentleman, I was extremely thankful for so plain a set of symptoms. All Hahnemannians know with what confidence I poured a few pellets on his tongue from my bottle of Eupat-perfol.$^{cm}$. That was nine days ago, and he has taken nothing but S. L. since that dose of Eupat-perfol. Neither has he had any more chills.

Just such grand results encourage me in my determination to practice pure Homœopathy.

Case III.—Was called five miles into the country to see an old lady who was suffering with excruciating pains in right side of abdomen; most intense in region of right ovary. Moving was unbearable. She was lying on the *painful* side. Her lips were very dry, and she was drinking large quantities of water. I will say here that she had sent for a squirt-gun doctor, but he was out, and I went instead. She begged me to inject Morphine, but I *would not.* She had called in her friends to see her die, but she did not die. One dose of Bry.$^{72m}$, dry on the tongue. Before we could give her a dose of S. L. she was asleep—the first sleep for forty-eight hours. She woke after about three hours' good sleep. From that time she went on to rapid recovery. If five thousand Hahnemannians had to prescribe for that case there would have been five thousand prescriptions of Bryonia. If all the old-school doctors in the country had to prescribe for her there would have been no two alike, except, perhaps, they would all have squirted Morphine into her.

All our cases are not such grand successes, but when we fail we take the blame on ourselves and not lay it on Homœopathy, as so many do. I find grand old *similia similibus* equal to all emergencies, and the results are most gratifying, indeed.

